# A Rare Case of Reversible Pulmonary Hypertension Phenotype in a Child with Scurvy: Aetiologies Insights

**DOI:** 10.3390/reports9010044

**Published:** 2026-01-30

**Authors:** Mattia Pasquinucci, Luisa Bonsembiante, Sofia Mezzalira, Martina Locallo, Davide Meneghesso

**Affiliations:** 1 Department of Pediatrics, AULSS 7 Pedemontana-San Bassiano Hospital, 36061 Bassano del Grappa, Italy; luisa.bonsembiante@aulss7.veneto.it (L.B.); sofia.mezzalira@aulss7.veneto.it (S.M.); davide.meneghesso@aulss7.veneto.it (D.M.); 2 Department of Neuroscience, Rehabilitation, Ophthalmology, Genetics, Maternal and Child Health, IRCCS Istituto Giannina Gaslini, 16100 Genoa, Italy; 3 Department of Pediatrics, University of Padua, 35100 Padua, Italy

**Keywords:** pulmonary artery hypertension, scurvy, vitamin C deficiency, hyperhomocysteinaemia, iron depletion, hypoxia-inducible factors

## Abstract

**Background and Clinical Significance:** Scurvy, caused by chronic vitamin C deficiency, is re-emerging in Western countries, particularly among pediatric patients with highly selective diets. While its musculoskeletal and mucocutaneous manifestations are well-known, its association with pulmonary arterial hypertension (PAH) is rare and poorly understood. Ascorbic acid and iron are essential cofactors for prolyl hydroxylases (PHD), which regulate Hypoxia-Inducible Factors. Their combined deficiency may trigger a “pseudohypoxic” state, leading to pulmonary vascular remodeling and vasoconstriction. **Case Presentation:** A 30-month-old female presented with a one-month history of limping, lower limb pain, and gingival hypertrophy. Dietary history revealed an almost exclusive cow’s milk-based intake. Physical examination showed diffuse petechiae, pallor, and right knee edema. Laboratory findings confirmed scurvy (undetectable vitamin C), severe iron-deficiency anemia (Hb: 72 g/L; ferritin: 22 mcg/L; RDW: 30%), folate deficiency, and hyperhomocysteinemia. Notably, elevated copper and vitamin B12 levels suggested a state of metabolic dysregulation. Echocardiography revealed moderate PAH phenotype (estimated sPAP: 47–50 mmHg) and a hyperdynamic contractility. A “perfect storm” mechanism was hypothesized, involving iron–ascorbate-dependent PHD impairment, high-output state, and oxidative-stress-induced hepcidin dysregulation (suggested by elevated copper). Following intravenous vitamin C and multivitamin supplementation, pulmonary pressures normalized within one week. **Conclusions:** PAH phenotype in scurvy represents a reversible metabolic disruption of pulmonary vascular tone rather than a structural disease. This case underscores the synergistic role of vitamin C, iron, and folate in vascular homeostasis. Clinicians should maintain high suspicion for scurvy in children with selective diets and unexplained PAH, as nutritional restoration is curative.

## 1. Introduction and Clinical Significance

Scurvy is a disease of historical significance, traditionally known as “the disease of sailors.” It results from chronic ascorbic acid deficiency and remains relatively frequent, though underdiagnosed, in developing countries where access to a balanced diet is limited. However, its incidence has been increasing in Western countries in recent years [1]. While it can affect adults, the pediatric population is generally more susceptible. It typically presents with cutaneous, mucosal, and musculoskeletal signs; early diagnostic markers include gingival hypertrophy with bleeding, limping or refusal to walk, and lower limb pain [2]. In cases of malabsorption, global malnutrition, or highly selective diets, scurvy is often associated with other micronutrient deficiencies [3]. Historically, there was no established link between scurvy and cardiovascular manifestations.

Clinical Significance: Recently, however, increasing attention has been paid to this correlation, and associated pulmonary arterial hypertension (PAH) has been rarely reported [4,5,6,7]. Nevertheless, its true incidence in patients with scurvy and its exact etiology remains incompletely understood.

## 2. Case Presentation

**Figure 1:**
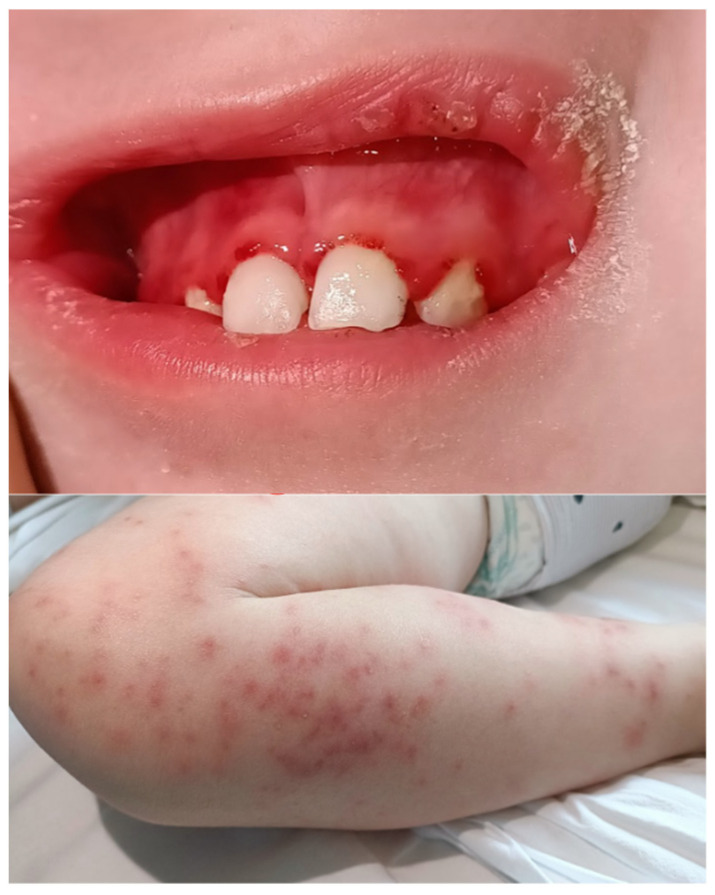
Haemorrhagic gingival hypertrophy and maculopapular rash on admission to the emergency department.

A 30-month-old female presented to our Emergency Department following several visits to other centers for a one-month history of monoarticular swelling, limping, and lower limb pain. The patient had sustained a fall approximately two weeks prior to the onset of symptoms. Clinical evaluation revealed periarticular edema and rubor of the right knee, a maculopapular rash on the same limb, diffuse petechiae, haemorrhagic gingival hypertrophy, and pallor (Figure 1).

Vital signs showed tachycardia and a systemic blood pressure of 90/55 mmHg (below normal limits). Dietary history disclosed a selective cow’s milk-based intake, accounting for over 95% of the child’s caloric intake.

Laboratory findings revealed severe hypochromic microcytic anemia without thrombocytopenia but with severe iron deficiency, along with mild coagulation abnormalities (increased Antithrombin III). Renal and hepatic functions were within normal limits, while electrolytes showed mild hyponatremia (Na^+^ 132 mmol/L). The metabolic–nutritional profile indicated global malnutrition with low triglycerides and HDL cholesterol and reduced prealbumin; blood glucose was normal. The initial differential diagnosis included malignant lymphoproliferative disease, which was reasonably excluded via peripheral blood smear; the latter instead showed neutrophil hypersegmentation (consistent with malnutrition/folate deficiency). Further biochemical analysis revealed low folate levels and increased homocysteine. High levels of D-dimer, copper, and vitamin B12 were also noted, initially interpreted as markers of systemic hyperinflammation (Table 1).

**Figure 2:**
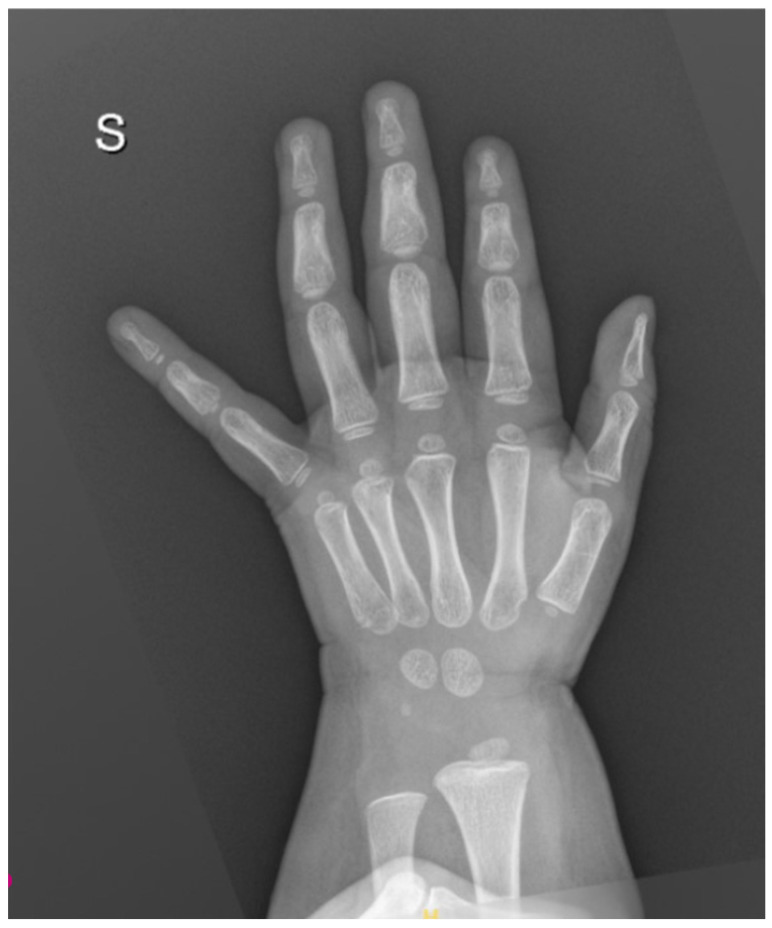
Anteroposterior view of the wrist showing slightly delayed bone age (bone age: 24 months according to Greulich and Pyle; chronological age: 30 months).

**Figure 3:**
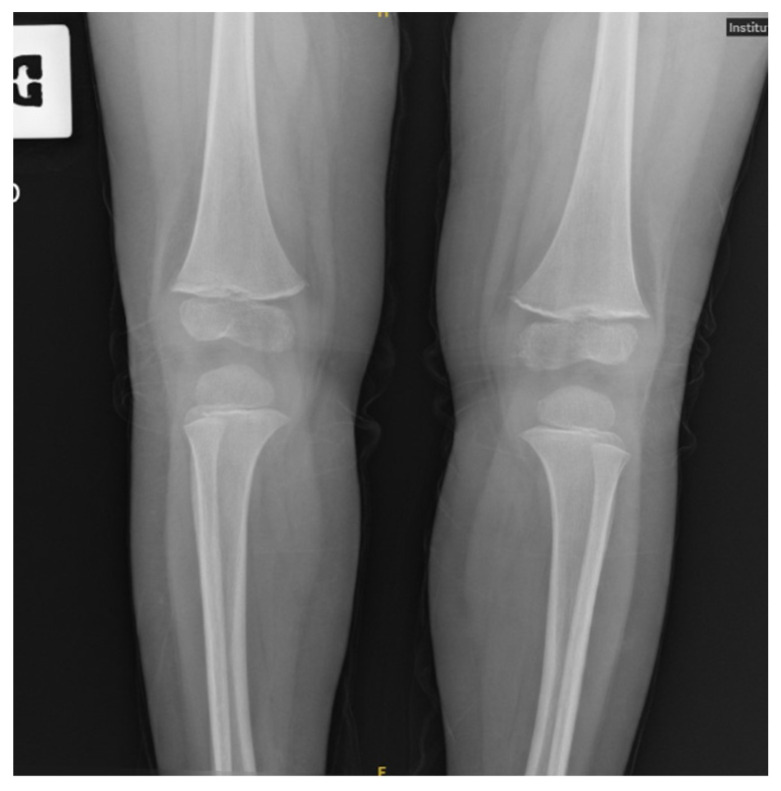
Imaging reveals pathognomonic skeletal changes in infantile scurvy. Key findings include the Trümmerfeld zone (a radiolucent band proximal to the dense zone of provisional calcification, representing a site of microfractures and disorganized matrix) and the white line of Fraenkel. Additionally, mild Pelkan spurs are visible at the metaphyseal margins.

The absence of significant lactic acidosis rendered a concomitant Thiamine deficiency less likely. Celiac disease was ruled out. Vitamin C was undetectable, confirming the diagnosis of scurvy. X-rays of the wrist showed slightly delayed bone age (Figure 2), while knee X-rays revealed typical skeletal changes pathognomonic for scurvy (Figure 3).

**Figure 4:**
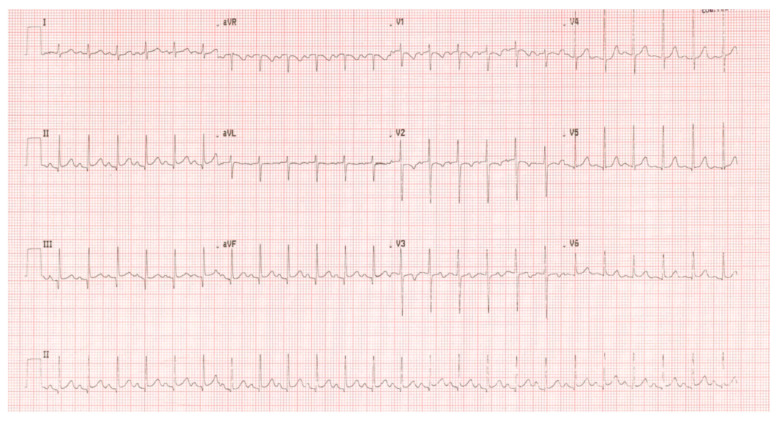
Twelve-lead electrocardiogram on admission, showing sinus tachycardia with mild alterations of the ventricular repolarisation phase (DII, DIII, avF), which were no longer evident after approximately one month of adjunctive therapy, reflecting the myocardial metabolic stress associated with global malnutrition and severe anemia.

Given the severity of the malnutrition, a cardiological evaluation was performed to rule out malnutrition-related cardiomyopathy before initiating high-volume fluids. Electrocardiography (ECG) showed diffuse repolarization abnormalities (Figure 4).

**Figure 5:**
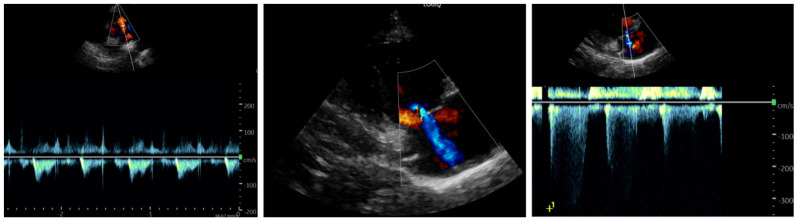
Findings suggestive of pulmonary hypertension can be observed, including midsystolic notching of the pulmonary artery flow, moderate tricuspid regurgitation, a measurable tricuspid regurgitant jet >2.8 m/s with an estimated systolic pulmonary artery pressure (sPAP) of approximately 47−50 mmHg (tricuspid regurgitation velocity: 42−45 mmHg; right atrial pressure: 5 mmHg) in, and slight end-diastolic flattening of the interventricular septum in the PSAX

Echocardiography, performed under midazolam sedation due to psychomotor agitation (but with stable hemodynamics), showed findings suggestive of PAH, including midsystolic notching of the pulmonary artery flow, moderate tricuspid regurgitation, a measurable tricuspid regurgitant jet >2.8 m/s with an estimated systolic pulmonary artery pressure (sPAP) of approximately 47–50 mmHg, assuming tricuspid regurgitation velocity 42–45 mmHg and right atrial pressure 5 mmHg, and slight end-diastolic flattening of the interventricular septum (Figure 5). The left heart appeared hyperdynamic. Despite these findings, the patient remained hemodynamically stable with normal resting oxygen saturation.

**Figure 6:**
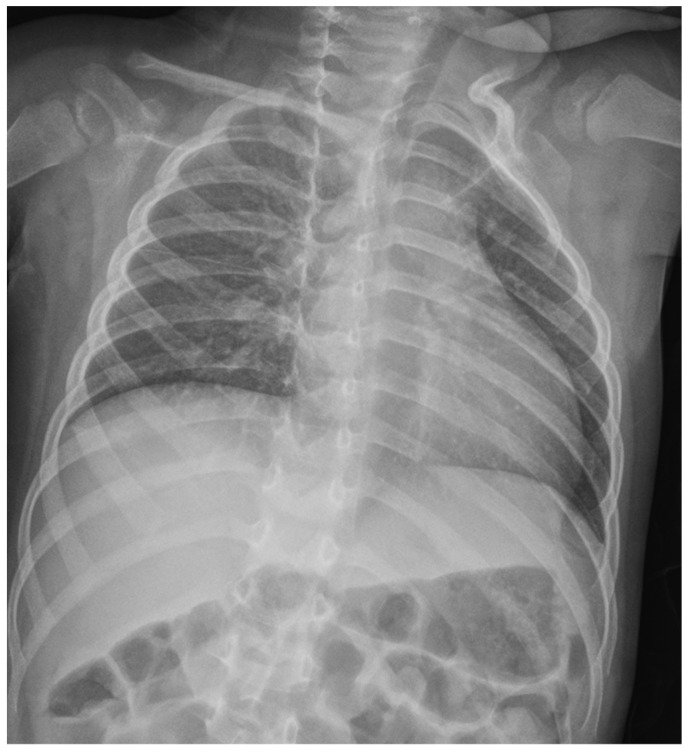
Anteroposterior chest X-ray at admission. The lung fields are clear with normal vascular markings and no evidence of parenchymal consolidation or pleural effusion. The cardiac silhouette is within normal limits for age.

Chest X-ray was performed to screen for pleuroparenchymal involvement or gross cardiomegaly (Figure 6). The exam revealed clear lung fields with normal vascular markings and a cardiac silhouette within the normal limits for age. These findings were instrumental in excluding primary lung diseases (Group 3 PH) and pulmonary venous congestion, thereby supporting the hypothesis of a functional, pre-capillary pulmonary hypertension phenotype.

Suspecting mixed malnutrition (deficiencies in vitamin C, vitamin K, and B-complex vitamins), intravenous vitamin C and multivitamin supplementation were initiated (Table 2).

**Figure 7:**
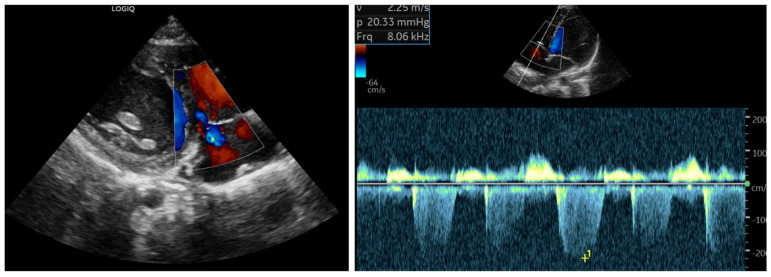
Follow-up echocardiography (7 days post−treatment): complete resolution of the midsystolic notching and normalization of the estimated systolic pulmonary artery pressure (<25 mmHg) following one week of intravenous vitamin C and multivitamin supplementation.

Follow-up ECG and echocardiography demonstrated complete normalization of pulmonary pressures after one week (Figure 7), which was confirmed one month after discharge along with the resolution of laboratory abnormalities.

The rapid reversibility of the PAH phenotype was documented through sequential echocardiographic assessments. A comprehensive comparison of the hemodynamic and functional parameters from admission to the 7th day of treatment is provided in Table 3.

**Figure A1:**
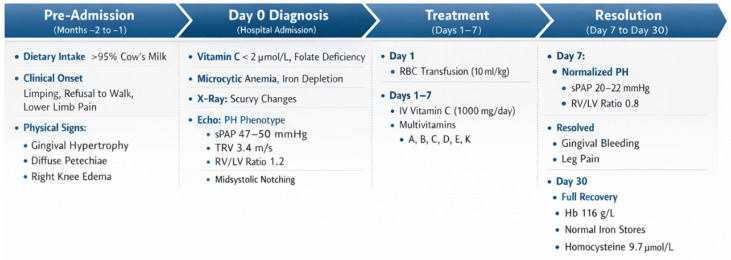
The sequence illustrates the progression from chronic dietary restriction (almost exclusive cow’s milk intake) to the symptomatic presentation of infantile scurvy. The diagnostic “perfect storm” at admission is marked by severe anemia, undetectable vitamin C levels, and a significant pulmonary hypertension phenotype. The timeline concludes with the rapid normalization of clinical signs and pulmonary pressures (within 7 days) following targeted vitamin C and multi-nutrient supplementation.

For a comprehensive overview of the clinical sequence—spanning from dietary habits and symptom onset to the resolution of the PH phenotype following treatment—please refer to the detailed timeline provided in Appendix A (Figure A1).

## 3. Discussion

PAH in scurvy has been rarely described, and it has been defined as pre-capillary (PVRi > 2 WU) in rare cases when cardiac cateterism was performed [8]. Table 4 summarizes the most frequent causes of PAH and their diagnostic tools that we used for differential diagnosis in the absence of a right heart cateterism.

Its pathophysiology in the context of scurvy is likely to be recognized as a multifactorial “perfect storm,” where acute vitamin C depletion acts as the final trigger on a pre-existing substrate of profound metabolic and nutritional derangement [9].

As established in previous reports, the most compelling mechanism involves the impairment of the endothelial–vascular homeostasis. Ascorbic acid and ferrous iron (Fe^2+^) are essential cofactors for prolyl hydroxylases (PHD), enzymes responsible for the degradation of Hypoxia-Inducible Factors (HIFs) [10,11]. In our patient, the simultaneous absence of vitamin C and severe iron deficiency (suggested by ferritin of 22 mcg/L and transferrin saturation of 5%) likely led to the pathological stabilization of HIF-1α and HIF-2α [12]. This “pseudohypoxia” state promotes pulmonary vasoconstriction and vascular remodeling, mimicking the effects of chronic altitude exposure despite normal oxygen saturation.

Building upon this established framework, we propose a further layer of complexity involving a disrupted iron-trafficking system. Although the C-reactive protein (CRP) was only mildly elevated (1.5 mg/dL), the scurvy-induced state of massive oxidative stress may have stimulated hepcidin production through non-canonical, ROS-dependent pathways. Elevated hepcidin levels, potentially reinforced by iron sequestration via lactoferrin in hypertrophic gingival tissues, would degrade ferroportin, leading to functional iron deficiency. In this framework, the observed elevation in serum copper (in the absence of significant systemic inflammation) becomes highly significant. It may reflect a compensatory, albeit futile, up-regulation of ceruloplasmin (a copper-dependent ferroxidase) in a desperate attempt to mobilize intracellular iron stores [13]. In our hypothesis, this “metabolic lock” would further starve the pulmonary vasculature of the iron necessary for PHD function, reinforcing the hypertensive cycle.

A different explanation may lie beyond systemic inflammation. The observed hypercupremia may be interpreted through the lens of impaired copper trafficking. As suggested by Harris et al., ascorbic acid is essential for labilizing copper bound to ceruloplasmin and facilitating its trans-membrane transport. In a state of profound scurvy, the absence of vitamin C may lead to a paradoxical accumulation of copper in the vascular compartment due to impaired cellular uptake, potentially exacerbating the functional iron deficiency and the subsequent HIF-mediated pulmonary vasoconstriction [14].

The finding of homocysteine levels twice the upper limit of normal serves as a crucial metabolic marker of this global derangement. While primarily interpreted as a signature of folate deficiency (consistent with the high RDW of 30% and neutrophil hypersegmentation), we suggest that hyperhomocysteinemia may have acted as a direct vascular toxin [15]. By impairing nitric oxide bioavailability and inducing oxidative stress, it likely narrowed the vasodilatory reserve of the pulmonary bed, making it more susceptible to the hemodynamic stress of an anemia-induced high-output state.

## 4. Conclusions

The rapid normalization of pulmonary pressures within one week of multivitamin supplementation, well before the correction of anemia or iron stores, strongly suggests that the recovery of ascorbate-dependent nitric oxide production and the resetting of the PHD/HIF axis were the primary drivers of resolution. This case highlights that PAH phenotype in scurvy might be considered not as a fixed structural disease but as a reversible metabolic disruption of the pulmonary vascular tone. Clinicians should consider scurvy in the differential diagnosis of unexplained echo-suspected PAH in children with selective diets, emphasizing that the cure lies not in vasodilators, but in targeted nutritional restoration.

## Figures and Tables

**Table 1:** The table summarizes the main biochemical findings at admission and after one month of effective therapy with vitamin D. The Hb value at 7 days of therapy, after blood transfusion, is also reported. An important microcytic iron-deficiency anemia is highlighted, along with a significant increase in D-dimer and ATIII. Additionally, an increase in copper levels and vitamin B12, likely of inflammatory origin, is noted, with a deficiency in folates and vitamin C. All biochemical findings normalized after approximately one month of therapy. Hb = hemoglobin; PT = Prothrombin Time; aPTT = Activated Partial Thromboplastin Time; ATIII = Antithrombin III.

Variable	On Admission	7th Day	30th Day	Reference Values
Hb (g/L)	72	95	116	107–144
MCV (fL)	63.6	73.1	82.5	80–95
RDW (%)	30	25	17.5	<15
Serum iron (mcg/dL)	32		57	16–128
Transferrin saturation (%)	5		11	10–43
Ferritin (mcg/L)	22.1		35.1	4.6–204.0
PT ratio	1.18		1.15	0.8–1.20
aPTT ratio	0.74		0.91	0.8–1.20
D-Dimer (ug/L)	4956	3765		<500
ATIII (%)	135		124	80–125
Vitamin B6 (nmol/L)	192			51–183
Vitamin B9 (mcg/L)	1.70		8.3	3.1–20.0
Vitamin B12 (ng/L)	1035		666	187–883
Vitamin C (umol/L)	<2		28	23–114
Vitamin A (nmol/L)	1053			800–2600
Vitamin E (umol/L)	15			10.1–21.0
Homocysteine (umol/L)	23.8		9.7	4.4–13.6
Serum copper (umol/L)	27.9		17.4	14.1–25.1
Lactate (mmol/L)	<2		<2	<2

**Table 2:** This table summarizes the main integrative therapies administered to the patient, along with their corresponding dosages and duration.

**Treatment**	**Posology**	**Duration**
Vitamin C iv	1000 mg/die (80 mg/kg/die)	7 days
Followed by Vit C po	300 mg/die (24 mg/kg/die)	ongoing (60 days)
Vitamin B 6 po	4 mg/die	ongoing (60 days)
Vitamin B 9 po	275 mcg/die	ongoing (60 days)
Vitamin B12 po	7 mcg/die	ongoing (60 days)
Vitamin A po	3 mg/die	ongoing (60 days)
Vitamin D po	500–1000 UI/die	ongoing (60 days)
Vitamin E po	40 mg/die	ongoing (60 days)
Vitamin K im	2 mg/week	2 weeks
Red blood cell transfusion	10 mL/kg	1st day

**Table 3:** Comparison of echocardiographic parameters at admission and after 7 days of treatment.

Echocardiography	At admission	Follow-Up (7th Day)
Systolic PAP (mmHg)	47−50	20–22
Tricuspidal regurgitation (m/s)	3.40	2.25
TAPSE (mm)	11	14
TAPSE/sPAP (mm/mmHg)	0.22	0.56
Midsystolic Notching	Present	Absent
RV/LV ratio	1.2	0.8
LVEF	75%	65%

**Table 4:** Systematic differential diagnosis workup for the observed echo-suspected pulmonary hypertension (PH) phenotype, illustrating the exclusion of primary cardiac, pulmonary, and thromboembolic etiologies.

Classification	Subgroup	Diagnostic Tool	Reason for Exclusion
Group 1	Congenital heart disease	Echocardiography	Normal cardiac anatomy
Group 1	Idiopathic or heritable	Clinical course	Immediate resolution after nutritional therapy
Group 2	Left heart disease	Echocardiography	No valvular disease
Group 3	Chronic lung disease or alveolar hypoxia	Chest X-ray O_2_ saturation	Clear lung fields on CXR; stable resting O_2_ saturation
Group 4	Pulmonary artery obstructions	D-dimer clinical course	Rapid resolution without anticoagulation
Group 5	Miscellaneous (nutritional)	Biochemicals	Normal lactate; no metabolic acidosis; undetectable Vit. C

## Data Availability

The raw data supporting the conclusions of this article will be made available by the authors on request.

## Abbreviations

The following abbreviations are used in this manuscript:

PAH	Pulmonary artery hypertension
ECG	Electrocardiography
sPAP	Systolic pulmonary artery pressure
PHD	Prolyl hydroxylases
HIFs	Hypoxia-Inducible Factors
CRP	C-reactive protein
RDW	Red cells distribution width
